# Implementing Electronic Discharge Communication Tools in Pediatric Emergency Departments: Multicountry, Cross-Sectional Readiness Survey of Nurses and Physicians

**DOI:** 10.2196/46379

**Published:** 2023-10-11

**Authors:** Janet Curran, Lori Wozney, Emma Tavender, Catherine Wilson, Krista C Ritchie, Helen Wong, Allyson Gallant, Mari Somerville, Patrick M Archambault, Christine Cassidy, Mona Jabbour, Rebecca Mackay, Amy C Plint

**Affiliations:** 1 IWK Health Centre Halifax, NS Canada; 2 Department of Psychiatry Dalhousie University Halifax, NS Canada; 3 Pediatric Emergency Research Canada Alberta Children's Hospital Calgary, AB Canada; 4 Clinical Sciences Murdoch Children’s Research Institute Melbourne Australia; 5 Department of Critical Care University of Melbourne Victoria Australia; 6 Paediatric Research in Emergency Departments International Collaborative Murdoch Children’s Research Institute Melbourne Australia; 7 Faculty of Education Mount Saint Vincent University Halifax, NS Canada; 8 Département de médecine familiale et médecine d'urgence Université Laval Québec City, QC Canada; 9 Department of Pediatrics University of Ottawa Ottawa, ON Canada; 10 Division of Emergency Medicine Children’s Hospital of Eastern Ontario Ottawa, ON Canada; 11 Department of Emergency Medicine University of Ottawa Ottawa, ON Canada

**Keywords:** discharge communication, pediatric, emergency department, medical informatics, implementation science, electronic medical record, mobile phone

## Abstract

**Background:**

Pediatric emergency departments (ED) in many countries are implementing electronic tools such as kiosks, mobile apps, and electronic patient portals, to improve the effectiveness of discharge communication.

**Objective:**

This study aimed to survey nurse and physician readiness to adopt these tools.

**Methods:**

An electronic, cross-sectional survey was distributed to a convenience sample of currently practicing ED nurses and physicians affiliated with national pediatric research organizations in Canada, Australia, and New Zealand. Survey development was informed by the nonadoption, abandonment, scale-up, spread, sustainability framework. Measures of central tendency, and parametric and nonparametric tests were used to describe and compare nurse and physician responses.

**Results:**

Out of the 270 participants, the majority were physicians (61%, 164/270), female (65%, 176/270), and had 5 or more years of ED experience (76%, 205/270). There were high levels of consensus related to the value proposition of electronic discharge communication tools (EDCTs) with 82% (221/270) of them agreeing that they help parents and patients with comprehension and recall. Lower levels of consensus were observed for organizational factors with only 37% (100/270) agreeing that their staff is equipped to handle challenges with communication technologies. Nurses and physicians showed significant differences on 3 out of 21 readiness factors. Compared to physicians, nurses were significantly more likely to report that EDs have a responsibility to integrate EDCTs as part of a modern system (*P*<.001) and that policies are in place to guide safe and secure electronic communication (*P*=.02). Physicians were more likely to agree that using an EDCT would change their routine tasks (*P*=.04). One third (33%, 89/270) of participants indicated that they use or have used EDCT.

**Conclusions:**

Despite low levels of uptake, both nurses and physicians in multiple countries view EDCTs as a valuable support to families visiting pediatric ED. Leadership for technology change, unclear impact on workflow, and disparities in digital literacy skills require focused research effort.

## Introduction

A staggering number of discharge communication interactions occur each year among health care providers, caregivers, and patients visiting pediatric emergency departments (ED). Over 3.1 million children and youths attended an ED for care in Canada between 2019 and 2020 with the majority (86%) discharged home [1]. Similarly, more than 1.6 million children visited Australian EDs in 2019-20, comprising 19.5% of national ED visits resulting in over 250,000 acute hospital admissions [2]. A review of 30 studies across 10 countries showed between 12% and 65% (mean 41.06, SD 15.16) of these visits are nonurgent presentations [3].

Throughout these visits, discharge communication processes play a vital role in helping caregivers and patients learn about the treatments received, gain the necessary knowledge and skills to continue care at home, ask questions, and receive instruction on symptoms that should prompt a return to the ED [4-6]. Health care organizations are progressively implementing more electronic discharge communication tools (EDCTs) such as computer kiosks, mobile apps, patient portals, and automated text message reminders to improve discharge communication interactions [7,8]. Patients report generally high satisfaction with these tools as part of the discharge process [9]. However, EDs are fast-paced, highly stressful, and highly distracting environments for engaging in discharge communication across a complex range of clinical presentations [4]. What might work to support discharge communication in another health sector (eg, primary care physicians’ use of a patient portal to share lab results), may not translate into effective practice in the ED context. Introducing a new EDCT may not merely accelerate or augment existing communication, but it may qualitatively restructure the discharge process as a whole [10]. Thus, successful implementation of EDCTs in the ED requires minimizing unintended negative consequences through appropriate readiness planning [11,12].

There is value in deepening understanding about the interplay between technology-related readiness indicators and broader organizational and system enablers [13]. Empirically applied across a range of health technology projects, the NASSS (nonadoption, abandonment, scale-up, spread, sustainability) framework provides a theory-driven lens to explore the uncertainties and interdependencies of unfolding technological initiatives [14]. This study aimed to leverage the NASSS framework and help identify factors that impact nurse and physician readiness to adopt EDCTs in pediatric EDs.

## Methods

### Study Design and Population

An electronic, cross-sectional, and self-administered survey was administered to a convenience sample of ED sites in 3 countries (Canada, Australia, and New Zealand). The survey and the study protocol were reviewed and approved by Pediatric Emergency Research Canada (PERC), Translating Emergency Knowledge for Kids (TREKK), and the Paediatric Research in Emergency Departments International Collaborative (PREDICT) network in Australia and New Zealand.

### Ethical Considerations

The study received ethical approval from IWK Health’s Research Ethics Boards in Canada (REB #1024535) and The Royal Children’s Hospital in Australia (HREC 2019.259). Informed consent was obtained from all participants.

### Inclusion Criteria

To participate, nurses and physicians were required to (1) be literate in English, (2) have access to email and internet, and (3) be a licensed nurse or physician currently working in an ED in Canada, Australia, or New Zealand. We aimed to recruit a minimum of 100 participants.

### Survey Content and Administration

The survey was developed by coauthors including ED physicians and nurses, family advocates, experts in psychometrics, implementation scientists, digital health developers, policy makers, and discharge communication researchers. To reduce the burden, only a select number of demographic questions were asked (eg, role, years in the ED, number of shifts per month, gender, ED site, and computer proficiency and confidence). Using the NASSS framework’s 7 domains as a guide (see Textbox 1), we generated 3 readiness-related questions for each domain. The 21 items were presented with a 5-point Likert scale of agreement: strongly disagree, disagree, neutral, agree, and strongly agree. Items 5 and 17 were negatively worded; thus, the interpretation of responses took that into account. Finally, the survey asked if an EDCT was currently in use in their ED and provided an open-text field to describe the EDCT features.

**Electronic discharge communication tool implementation readiness survey items related to NASSS (nonadoption, abandonment, scale-up, spread, sustainability) domains.**

**Domain 1: complexity in the illness or conditions being treated in the environment where the technology is used**

1. There are standardized education and discharge instructions for most families who visit our emergency department (ED)

2. The diversity of families (eg, language, cultural practices and health literacy levels) visiting our ED poses significant challenges for standardized discharge communication

3. The use of an electronic discharge communication tool is suitable for our ED setting

**Domain 2: complexity in the features of the technology itself**

4. Data generated by electronic discharge communication tools can inform clinical practice

5. Our ED technology environment (eg, Wi-Fi connection, access to computers, printers, or other technologies) is unreliable (items are negatively worded)

6. Patient care can be improved with an effective electronic discharge communication tool

**Domain 3: value proposition of the technology**

7. Most families (eg, 75%) who visit our ED have access to at least 1 personal electronic device (eg, smartphone, computer, and tablet)

8. There is value in using an effective electronic discharge communication tool in our ED

9. Our ED has a responsibility to integrate effective electronic communication tools as part of a modern health care system

**Domain 4: capacity or willingness of the end user to adopt the technology**

10. Access to technology support is important for our ED team to use an electronic discharge communication tool

11. The use of an electronic discharge communication tool would change my routine tasks

12. An electronic discharge communication tool would help parents and patients with comprehension and recall of information given in the ED

**Domain 5: whether organizational constraints, such as budgets and infrastructure were taken into consideration**

13. Our organization values the use of electronic tools by dedicating sufficient budget

14. Leadership in our ED manages technology-related change well

15. Our organization provides timely technical assistance to ED staff who use electronic tools in their work activities

**Domain 6: complexity within the broader systems and context features such as professional guidelines, policies, and regulatory factors**

16. I am concerned about the regulatory and legal requirements of using electronic communication tools in my workplace

17. My professional licensing body is not supportive of electronic communication with patients and parents (items are negatively worded)

18. Policies and practice guidelines are in place to guide safe and secure electronic communication with patients and parents

**Domain 7: necessity of a technology to be flexible over time in order to adapt to changes within the system**

19. Our ED staff is equipped to handle challenges with communication technologies (eg, Wi-Fi connection unavailable)

20. Our ED team is capable of adapting to challenges resulting from the technology

21. There is an urgency to routinely use evidence-based electronic communication tools in our ED

The survey was pilot-tested with 3 ED clinicians and made available in English and French. Slight modifications to the wording were made for the version sent to Australian and New Zealand clinicians to ensure alignment with local conventions (eg, labeling current role options as Fellow of the Royal Australasian College of Physicians instead of Certification in the College of Family Physicians as was used on the Canadian survey). The web-based consent form and survey were hosted on the Research Electronic Data Capture (REDCap) [15] platform and took approximately 5 minutes. Consent was implied from survey completion.

The survey was administered in Canada between November 2019 and February 2020 and in Australia and New Zealand between December 2019 and February 2020. To recruit Canadian participants, an email was sent to all members of the PERC Survey Database of Physicians with an active email address (n=211). Site coordinators or representatives for PERC were invited to send the link to a convenience sample of 8-10 nurses in their ED. In addition, the survey was distributed by the Director of the TREKK network to physician representatives from 37 general ED TREKK sites across Canada. To recruit Australian and New Zealand participants, an email invitation was sent to physician and nurse members (n=121) of the PREDICT network by the network coordinator (CW). A modified Dillman method [16] was applied to maximize response rates so site coordinators were asked to send 2 email reminders within 3 months. Participant responses were anonymized prior to analysis.

### Data Analysis

Descriptive statistics were performed using the open-source platform JASP (version 16; Jeffreys's Amazing Statistics Program) [17] to summarize measures of central tendency. Participant characteristics were compared using the chi-square test, *t* test, or Mann-Whitney *U* test. Differences were considered statistically significant at *P*<.05, and all tests were 2-tailed. Text responses to open-ended response items were exported into an Excel (Microsoft Corp) spreadsheet and inductive content analysis [18] was performed.

## Results

### Demographics

A total of 270 ED clinicians completed the survey (n=164 physicians; n=106 nurses). There were 231 participants from Canada and 39 combined from Australia and New Zealand. We were not able to calculate an exact response rate for Canadian sites but there was at least 1 nurse or physician respondent from each PERC site in Canada, 6 respondents from TREKK sites, and an overall 32% response rate among Australian or New Zealand sites. No significant difference was noted between Canadian and Australian or New Zealand groups in terms of years of work in the ED (*χ*^2^_3_=9.4; *P*=0.03), gender (*χ*^2^_2_=4.4; *P*=.11), number of monthly shifts (*χ*^2^_3_=0.05; *P*>.99), current use of an EDCT (*χ*^2^_2_=1.5; *P*=.48), computer proficiency (*t*_264_=–1.467; *P*=.14), or level of confidence in learning new technologies (*t*_264_=–0.755; *P*=.45). There were no significant differences between countries on any of the 21-NASSS items, therefore data were pooled for analysis. Demographic characteristics of participants can be found in Table 1.

**Table 1:** Characteristics of participating physicians and nurses.

Participating		Physicians (n=164)	Nurses (n=106)	All participants (n=270)
**Gender, n (%)**				
	Male	84 (51.2)	4 (3.7)	88 (32.6)
	Female	75 (45.7)	101 (95.3)	176 (65.2)
	Prefer not to say	5 (3.1)	1 (0.9)	6 (2.2)
**Country of practice, n (%)**				
	Canada	137 (83.5)	94 (88.7)	231 (85.6)
	Australia or New Zealand	27 (16.5)	12 (11.3)	39 (1.4)
**Language, n (%)**				
	English	152 (92.7)	106 (100.0)	258 (95.6)
	French	12 (7.3)	0 (0)	12 (4.4)
**Years in ED^a^ practice, n (%)**				
	<5	24 (14.6)	41 (38.7)	65 (24)
	5 to 10	40 (24.3)	20 (18.9)	60 (22)
	11 to 20	65 (39.6)	30 (28.3)	95 (35)
	>20	35 (21.3)	15 (14.2)	50 (18)
**Monthly ED shifts, n (%)**				
	1-4	23 (14.0)	8 (7.5)	31 (11.5)
	5-8	40 (24.4)	14 (13.2)	54 (20.0)
	9-12	55 (33.5)	24 (22.6)	79 (29.3)
	>12	46 (28.0)	60 (56.6)	106 (39.3)
**Currently use electronic discharge tool, n (%)**				
	Yes	58 (35.4)	31 (29.2)	89 (33.0)
	No	106 (64.6)	58 (54.7)	164 (60.7)
	Missing	0 (0)	17 (16.1)	17 (0.1)
Proficiency with technology (1-100), mean (SD)		77.43 (15.3)	79.2 (14.2)	78.1 (14.9)
Confidence learning new computer skills (1-100), mean (SD)		81.4 (15.12)	84.5 (15.6)	82.6(15.4)

^a^ED: emergency departments.

Among physicians, 61% (100/164) had been working in the ED environment for 11 years or more. In contrast, only 42% (45/106) of nurses had worked in the ED for that length of time. In this, 39% (106/270) of participants were working more than 12 shifts a month in the ED. Overall, participants reported a relatively high level of proficiency with computer technologies (mean 78.14, SD 14.91); and confidence in learning new computer-related skills (mean 82.62, SD 15.38). Two-thirds of the participants (61%, 164/270) were not using an EDCT in their ED practice at the time of survey response.

### NASSS Implementation Domains

**Figure 1:**
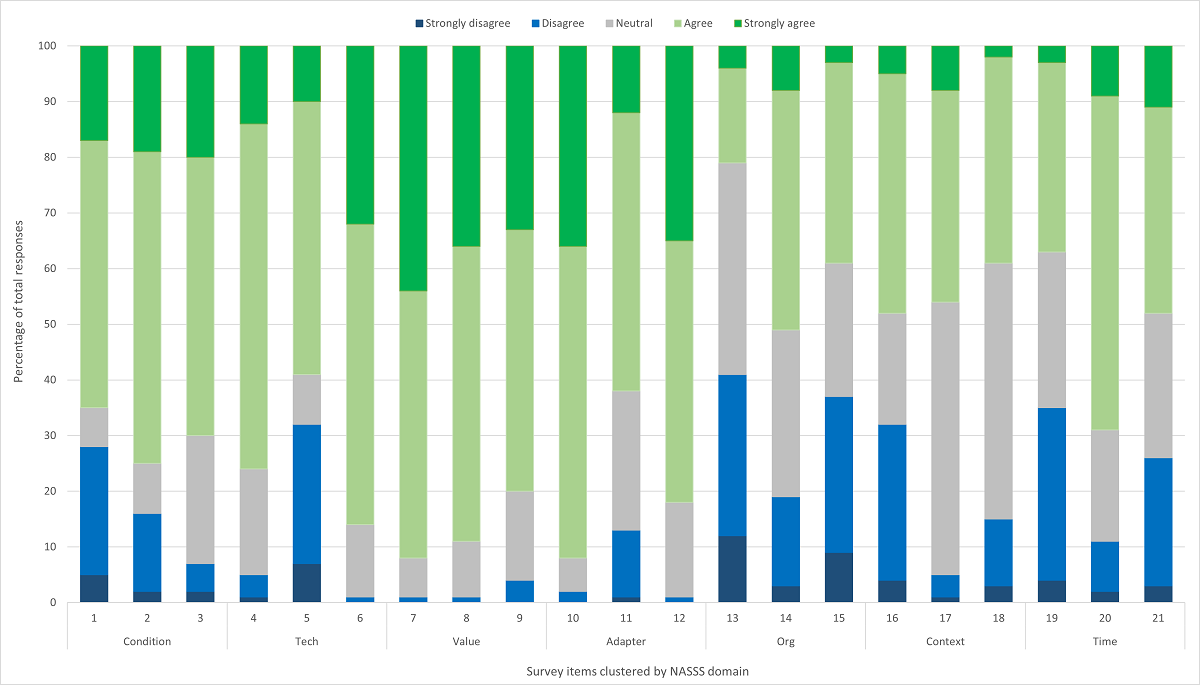
Percentage of agreement across readiness survey implementation domains and items. NASSS: nonadoption, abandonment, scale-up, spread, sustainability.

As shown in Figure 1, the vast majority (88%, 251/270) of the participants strongly agreed or agreed that there is value in using an effective EDCT in their ED (item 8) and that an EDCT would help parents and patients with comprehension and recall of information given in the ED (82%, 222/270; item 12). The NASSS domain with the overall strongest level of item agreement (ie, endorsed most by agree or strongly agree for all 3 items) was domain 3 (value proposition). In this, 92% (248/270) of participants agreed that families have access to a personal electronic device (item #7), 88% (251/270)agreed that there is value in using EDCTs (item 8), and 80% (195/270) agreed that their ED has a responsibility to integrate effective electronic communication tools as part of a modern health care system (item 9). Despite perceiving EDCTs as having a high value, 75% (204/270) of the participants agreed or strongly agreed that the diversity of families (eg, language, cultural practices, and health literacy levels) visiting their ED poses significant challenges for standardized discharge communication (item 2).

The NASSS domain where participants in our study responded as disagree or strongly disagree most often across all 3 items was domain 5 (organizational factors). In this, 41% (111/270) disagreed or strongly disagreed that sufficient budget is spent on electronic tools (item #13), 19% (70/270) disagreed or strongly disagreed that leadership in their ED manages technology-related change well (item 14), and 37% (137/270; disagreed or strongly disagreed that there is timely technical assistance to ED staff who use electronic tools in their work activities (item 15). The percentage of “neutral” responses ranged from 6% (16/270; item 10: access to technology support is important for our ED team to use an electronic discharge communication tool); to 50% (134/270; item 17: my professional licensing body is not supportive of electronic communication with patients and parents). While the majority of participants reported their ED teams were capable of adapting to challenges resulting from technology over time (69%, 186/270; item 20, domain 7), only 36% (97/270) agreed or strongly agreed that their staff is equipped to handle challenges with communication technologies (item 19, domain 7). It is possible gaps in confidence with leadership play a role considering only half of the participants (50%, 136/270) agreed or strongly agreed that their leaders manage technology well (item 14).

Due to the skewness of data, Mann-Whitney *U* tests were conducted to analyze differences between physicians and nurses among the 21-implementation items. Physicians and nurses generally agreed on 86% (18/21) of implementation items. As outlined in Table 2, the test revealed significant differences between 3 items, each from a different NASSS domain. Compared to physicians, nurses were significantly more likely to report that EDs have a responsibility to integrate EDCTs as part of a modern system (*P*<.001) and that policies are in place to guide safe and secure electronic communication (*P*=.02). Physicians were more likely to agree that using an EDCT would change their routine tasks (*P*=.04).

**Table 2:** Significant differences between physicians and nurses.

Item (#, domain) and care provider		Mean (SD)		*W*		*P* value	
**Our emergency departments has a responsibility to integrate effective electronic communication tools as part of a modern health care system (9, 3)**					6537.5		<.001
	Physicians	3.94 (0.85)					
	Nurses	4.30 (0.71)					
**The use of an electronic discharge communication tool would change my routine tasks (11, 4)**					9723.0		.04
	Physicians	3.70 (0.82)					
	Nurses	3.45 (0.95)					
**Policies and practice guidelines are in place to guide safe and secure electronic communication with patients and parents (18, 6)**					7239.0		.02
	Physicians	3.14 (0.80)					
	Nurses	3.39 (0.76)					

### Current Use of EDCTs

One-third of the participants (85/270) provided descriptions of the EDCT used in their ED in the open-ended survey question. Content analysis showed that 93% (79/85) of those descriptions identified Electronic Medical Record (EMR) systems as the EDCT in use, while 4% (3/85) were websites, 2% (2/85) were videos, and 2% (2/85) were electronic forms or fillable PDFs. A typical description of how an EMR was used as an EDCT involved a health care provider inputting information (eg, electronic health record, after-visit summary) and it auto-generating a printable discharge summary report that was given to caregivers or patients prior to leaving the ED. In 4 instances (4/85) providers mentioned caregivers being able to access the report through an electronic patient portal.

None of the open-ended responses were coded as descriptions of enablers. However, a wide range of both patient-level and organizational or environmental-level factors that may negatively impact implementation were reported.

A participant (1/85) noted uncertainty about the rate of patient sign-up for EDCTs and stated, “definitely think it is harder for refugee, low socioeconomic, indigenous and even certain ethnic populations to use or have access to our electronic tools.” Limited ability to translate into languages used by patients and families was cited by 4% (3/85) of participants. A participant (1/85) commented on how complex the medical information is for caregivers to understand: “The information that they received is both from a discharge form as well as test results, [this] was quite confusing to them. In essence...they needed me to interpret the information for them.”

Organizational and environmental level barriers were sometimes vaguely described as “not very good” and “not user friendly, our discharge summary completion rates are generally only 65%-70%” (2/85). Training-related concerns, such as having access to an EDCT but not having had the training to know how to use the tool, were the most frequently mentioned organizational barrier (4/85). Others included (1) impact on quality of communication “most [staff] do not use this tool as it doesn’t do a sufficient job of summarizing conversations had with health care professionals” (1/85), (2) lost confidence among staff due to recent cyber-attack in the ED (1/85), (3) concerns related to outdated technology being replaced (1/85), and (4) confusing hybrid approaches where ED staff engage in discharge communication using combinations of emailing, texting, and paper-based and verbal instructions (1/85).

## Discussion

### Overview

The aim of this study was to leverage the NASSS implementation framework in a novel way to identify emergent uncertainties and interdependencies that impact nurse and physician readiness to adopt EDCTs in pediatric EDs.

Across all study participants, there was strong consensus about the value of EDCTs. Clinicians reported high agreement with the impact of EDCTs on improving patient and caregiver comprehension and recall, supporting modern health care innovation, informing their clinical practice and the accessibility of digital devices among young families visiting the ED. This high level of consensus was observed despite a wide range of self-reported computer proficiency skills (range 25-100) and confidence levels in learning new technology (range 25-100).

Our findings suggest that while there may already be “buy-in” for EDCTS even from less technology-literate staff, their use is still not widespread [19-21]. Implementation efforts might benefit then, from focusing on environmental contextual factors rather than trying to change provider attitudes and beliefs. This result aligns with Canadian research exploring nurse adoption of other information systems [22].

While clinicians in our study see a positive value proposition for EDCTs there were mixed responses across other implementation domains, namely the organizational and societal contexts. Only 22% (59/270) of participants felt their organization valued digital technologies based on budget allocation, while about one-third of participants agreed that their organization offered timely support for technological challenges.

Differences between nurses and physicians were limited. Our survey revealed significant differences between those groups for only 3 of the 21-items and in all cases, it was the magnitude of difference, not the direction of opinion that was different. Open-ended responses reflected some concerns about caregivers’ preferences and skills in using EDCTs. Digital equity and its intersection with other racial and cultural disparities observed in ED [23] warrants future analysis within EDCT implementation studies. Given the wide range of implementation barriers reported at the patient and organization levels, additional qualitative research with this population may be helpful in reconceptualizing what we have learned so far and theorizing and generating new ways of exploring readiness [24].

Given EMRs were largely used to support discharge communication in clinician-driven (eg, clinicians complete data entry and prints and hand over paper copy) not patient-centered ways, future research should explore how patient-led and more interactive EDCTs (kiosks, mobile apps, bidirectional text messaging, and interactive websites) might support high-quality discharge communication in different ways or require different types of readiness to implement. In particular, examining the perspectives of youth and caregivers on their readiness to use these tools in an ED context would add significantly to the field.

### Limitations

A limitation of this study was the approach to sampling. Because participation was voluntary, those who chose to participate may have already held more positive attitudes about, or stronger interest in EDCTs and may be overrepresented in the sample. Additionally, representation across countries was not equal and future analysis should explore similarities and differences to reduce bias introduced by this overrepresentation.

### Conclusions

Nurse and physician readiness to integrate technologies into clinical pathways for discharge communication in pediatric ED is not only impacted by the availability of technology infrastructure. This multicountry study offers an original application of the NASSS framework to help emergency medicine leaders and administrators begin to systematically address the broader factors that are contributing to current low rates of uptake.

## Abbreviations

ED: emergency departments
EDCT: electronic discharge communication tool
EMR: Electronic Medical Record
NASSS: nonadoption, abandonment, scale-up, spread, sustainability
PERC: Pediatric Emergency Research Canada
PREDICT: Paediatric Research in Emergency Departments International Collaborative
REDCap: Research Electronic Data Capture
TREKK: Translating Emergency Knowledge for Kids
